# Upscaling of Electrospinning Technology and the Application of Functionalized PVDF-HFP@TiO_2_ Electrospun Nanofibers for the Rapid Photocatalytic Deactivation of Bacteria on Advanced Face Masks

**DOI:** 10.3390/polym15234586

**Published:** 2023-11-30

**Authors:** Adriano Cimini, Alessia Borgioni, Elena Passarini, Chiara Mancini, Anacleto Proietti, Luca Buccini, Eleonora Stornelli, Emily Schifano, Simone Dinarelli, Francesco Mura, Claudia Sergi, Irene Bavasso, Barbara Cortese, Daniele Passeri, Enrico Imperi, Teresa Rinaldi, Alfredo Picano, Marco Rossi

**Affiliations:** 1 Department of Basic and Applied Sciences for Engineering, Sapienza University of Rome, Via A. Scarpa 16, 00161 Rome, Italyanacleto.proietti@uniroma1.it (A.P.); luca.buccini@uniroma1.it (L.B.); eleonora.stornelli@uniroma1.it (E.S.); daniele.passeri@uniroma1.it (D.P.);; 2 Industrial Research Laboratory, LABOR s.r.l., Via Giacomo Peroni 386, 00131 Rome, Italy; 3 Department of Biology and Biotechnologies, Sapienza University of Rome, Piazzale Aldo Moro 5, 00185 Rome, Italy; borgioni.1858542@studenti.uniroma1.it (A.B.); elena.passarini@uniroma1.it (E.P.);; 4 Institute for the Structure of Matter (ISM), National Research Council (CNR), Via del Fosso del Cavaliere 100, 00133 Rome, Italy; simone.dinarelli@ism.cnr.it; 5 Research Center for Nanotechnology for Engineering of Sapienza (CNIS), Sapienza University of Rome, Piazzale Aldo Moro 5, 00185 Rome, Italy; 6 Department of Chemical Engineering Materials Environment, Sapienza University of Rome & UdR INSTM, Via Eudossiana 18, 00184 Rome, Italy; 7 National Research Council (CNR), Institute of Nanotechnology (CNR Nanotec), c/o Edificio Fermi, Sapienza University of Rome, Piazzale Aldo Moro 5, 00185 Rome, Italy; barbara.cortese@nanotec.cnr.it; 8 National Research Council of Italy, Institute for Microelectronics and Microsystems (CNR-IMM), Via Piero Gobetti 101, 40129 Bologna, Italy

**Keywords:** Electrospinning, photocatalytic antibacterial activity, antimicrobial air filter, personal protective equipment, nanofibers, electrospun

## Abstract

In recent years, Electrospinning (ES) has been revealed to be a straightforward and innovative approach to manufacture functionalized nanofiber-based membranes with high filtering performance against fine Particulate Matter (PM) and proper bioactive properties. These qualities are useful for tackling current issues from bacterial contamination on Personal Protective Equipment (PPE) surfaces to the reusability of both disposable single-use face masks and respirator filters. Despite the fact that the conventional ES process can be upscaled to promote a high-rate nanofiber production, the number of research works on the design of hybrid materials embedded in electrospun membranes for face mask application is still low and has mainly been carried out at the laboratory scale. In this work, a multi-needle ES was employed in a continuous processing for the manufacturing of both pristine Poly (Vinylidene Fluoride-*co*-Hexafluoropropylene) (PVDF-HFP) nanofibers and functionalized membrane ones embedded with TiO_2_ Nanoparticles (NPs) (PVDF-HFP@TiO_2_). The nanofibers were collected on Polyethylene Terephthalate (PET) nonwoven spunbond fabric and characterized by using Scanning Electron Microscopy and Energy Dispersive X-ray (SEM-EDX), Raman spectroscopy, and Atomic Force Microscopy (AFM) analysis. The photocatalytic study performed on the electrospun membranes proved that the PVDF-HFP@TiO_2_ nanofibers provide a significant antibacterial activity for both *Staphylococcus aureus* (~94%) and *Pseudomonas aeruginosa* (~85%), after only 5 min of exposure to a UV-A light source. In addition, the PVDF-HFP@TiO_2_ nanofibers exhibit high filtration efficiency against submicron particles (~99%) and a low pressure drop (~3 mbar), in accordance with the standard required for Filtering Face Piece masks (FFPs). Therefore, these results aim to provide a real perspective on producing electrospun polymer-based nanotextiles with self-sterilizing properties for the implementation of advanced face masks on a large scale.

## 1. Introduction

In the last few years with the pandemic outbreak, wearing face masks has been recognized by the Word Health Organization (WHO) as the main form of protection to prevent any risk of infection from coronavirus disease (COVID-19) [1,2,3]. The ability of the virus to coalesce with both large particles, ranging in size between ~2.5–10 μm, and smaller pollution ones, with diameters smaller than 1 μm, present in the atmosphere makes it extremely infectious. This is especially true for close-distance contacts, where the respiratory infection can be transmitted for a short period from an infected person to a healthy one during talking, coughing, and sneezing, and it can also remain suspended in the air for a long time, before spreading at longer distances [4,5,6]. In this perspective, commonly used face masks, such as surgical and filtering half-masks (i.e., Filtering Face Pieces (FFPs)), turned out to be essential barriers to protect the wearer from submicron particles and biological contaminants that can be present in the form of droplets or aerosols in the atmosphere, reducing the spreading of and infection by the coronavirus (SARS-CoV-2) [1,2,3,7]. In order to determine the effectiveness of these face devices in preventing users from being infected by large and small droplets, which can be potential bacteria or virus carriers, the European Norm (EN) specifies several performance filtration criteria, which include Bacterial Filtration Efficiency (BFE) and Particle Filtration Efficiency (PFE) [8,9,10]. However, Viral Filtration Efficiency (VFE) is an unrecognized standard parameter that is commonly employed to quantify the degree of protection of face devices from virus aerosols [11,12,13]. This test follows a similar procedure and setup to that recommended by EN 14683:2019 for BFE [9], where a bacterial suspension of *S. aureus* is employed, using instead a suspension of bacteriophages, such as ϕx174 and MS2, that are commonly aerosolized as nonhazardous virus surrogates for SARS-CoV-2 in laboratory experiments [14]. Despite the fact that most of the commercial face masks are expected to effectively remove submicron particles present in the surroundings, the filter materials used are not able to inactivate both bacteria and viruses, thus making the utilization of these face devices disposable and very unsafe in case they have been worn for a longer period than that recommended. An evident proliferation of fungi and bacteria were observed in common face masks, because of the moist air expired and inspired after prolonged use, thus representing a health risk especially in severe cases of illness [15,16,17,18,19]. Therefore, the disinfection performance of disposable masks has become a relevant issue from the start of the pandemic, in order to avoid the risk of further contamination in the long run caused by the improper and continuous accumulation of waste masks on the environment.

The layers forming a commercial filter media are mainly produced by means of the Melt-Blowing (MB) method and are generally composed of polypropylene (PP), i.e., a thermoplastic polymer, whose fiber diameters are about the submicron size [20]. Unlike MB, Electrospinning (ES) proved to be a promising technique to customize a broad range of synthetic and natural polymer-based electrospun membranes with fiber diameters and pore sizes down to the nanoscale, providing remarkable performance in both Particulate Matter (PM) capture and low pressure drop [8]. Furthermore, the implementation of active metal oxide Nanoparticles (NPs), such as ZnO [21], TiO_2_ [22], and metal Ag [23], as well as other bioactive hybrid nanocomposites, including aggregation-induced emission (AIE)-active photosensitizer [24], metal-organic frameworks (MFO) [25], and rose bengal (RB) [26] in polymer-based electrospun nanofiber filters, have proven to be advantageous to design environmentally friendly face masks with tailored filtration performance as well as photocatalytic properties, which make them able to deactivate both bacteria and viruses after an exposure to light sources, and thus extending the lifetime quality of the device and making it reusable [8]. The generation of Reactive Oxygen Species (ROS) induced at the nanofiber surface level by the irradiation of these embedded photocatalytic nanomaterials with visible–UV light involves a complete inactivation of bacteria and viruses by drastically affecting protein and enzyme function, due to the electrostatic interaction with the bacterial cell walls and membranes [27,28,29]. Although the first steps in designing hybrid photocatalytic materials for face mask applications based on ES have been reported in the literature, the number of published studies is still low, and the research has been mainly carried out at the laboratory scale using a conventional single nozzle. ES using a single nozzle provides a low-rate productivity of ~ 0.01–0.1 g/h compared to other standard MB setups that are commonly used for the manufacturing of commercial polymer-based filters on a large scale [8]. In order to address the issue of long fiber deposition time due to single-needle use, several configurations involving multi-needles and multi-nozzles have been developed over the years [30]. These setups are based on conventional ES, but with the utilization of a nozzle array that allows for the outflow of several polymer solutions simultaneously, thus involving a high productivity rate of the nanofibers [31,32,33].

In this work, a multi-nozzle ES setup for high throughput was employed to design a new photocatalytic nanofiber-based membrane filter based on Poly (Vinylidene Fluoride-*co*-Hexafluoropropylene) (PVDF-HFP) and decorated with TiO_2_ NPs. Among the active metal oxide compounds available, TiO_2_ NPs are commercially attractive for the manufacturing of UV–visible light self-sterilizing face masks because of their high photocatalytic bactericidal activity, chemical stability, low cost, and biocompatibility with several electrospun polymer mats [28,34,35]. The TiO_2_ anatase can be activated by means of the irradiation of light with energy higher than its band gap (~3.06 eV), thus promoting the formation of excited electron–hole pairs [29]. Furthermore, the optical performance of TiO_2_ NPs has been shown to remain quite stable and to provide photocatalytic activity in the UV range when also embedded in polymer-based electrospun membranes [22,36,37,38,39,40]. The Poly (Vinylidene Fluoride) PVDF proved to be an excellent polymer material support to implement TiO_2_ NPs as well as other active metal oxide nanomaterials on the electrospun-based nanofibers, by maintaining their optimal surface area and photocatalytic properties [41]. Compared to PVDF, the copolymer PVDF-HFP has better mechanical properties, as well as thermal and chemical stabilities, which make it promising for the manufacturing of advanced filters in applications for wearable healthcare devices [42,43]. In addition to the aforementioned properties, the PVDF and its fluorinated copolymer PVDF-HFP are widely implemented in health applications, including in personal protective clothing and tissue engineering, because of their biocompatibility characteristics [44,45,46,47,48,49,50,51]. The filter designed by electrospun PVDF-HFP nanofibers on Polyethylene terephthalate (PET) nonwoven spunbond layers achieved an optimal filtration performance against both *S. aureus* and bacteriophage ϕx174 virus aerosols. Additionally, the PVDF-HFP electrospun membrane functionalized with TiO_2_ NPs not only provides a high PM capture and optimal pressure drop that meet the standard for FFPs, but also bactericidal activity against both gram-positive *S. aureus* and gram-negative *P. aeruginosa* after a short exposure time to UV-A light radiation. *S. aureus* and *P. aeruginosa* were chosen as representatives of, respectively, gram-positive and gram-negative pathogens. They are responsible for severe infections that are challenging to treat because they are able to resist several antibiotics, making them particularly interesting to investigate [52,53,54]. Herein, we provide further insight into the production of PVDF-HFP@TiO_2_ nanofiber filter membranes by means of ES for the realization of advanced face masks, with a functionalized filter via the addition of photocatalytic TiO_2_ NPs material to the PVDF-HFP solutions that can enable an optimal antibacterial capability, thus minimizing the risk given by the handling and reuse of contaminated face masks.

## 2. Materials and Methods

### 2.1. Method for Fabrication of Nanofibers

The electrospun fibers were synthesized with the addition of PVDF-HFP (Kynar Flex 2801 Arkema Inc., Colombes, France) at 16.5 wt.%, with respect to the total mass solution, in N-Dimethylformamide (DMF, Sigma Aldrich, St. Louis, MO, USA, purity 99.8%) (62 wt.%) and Methyl Ethyl Ketone (MEK, Merck, Darmstadt, Germany) (21 wt.%). To make the dispersion of the anatase TiO_2_ NPs (<25 nm, Sigma-Aldrich, St. Louis, MO, USA) more homogenous, Cetyltrimethylammonium Bromide (CTAB, Sigma Aldrich, St. Louis, MO, USA) (0.45 wt.%) was first mixed in the solvent-based solution of DMF/MEK for 1 h and the TiO_2_ NPs at 2 wt.% were successfully added into it and stirred for 1 h. After that, the PVDF-HFP was slowly added and mixed in the precursor solution at a temperature of 60 °C until a homogeneous solution was obtained. The homogeneous solution was further loaded into the plastic syringe arrays to be electrospun at room temperature by means of a multi-nozzle NS24 ES system designed by INOVENSO (Co., Ltd., Istanbul, Turkey). This technology setup is a high-throughput machine for the lab-scale equipped with 12 nozzles that allows for obtaining a nanofiber coating area of about ~37 × 28 cm^2^. Both PVDF-HFP and PVDF-HFP @TiO_2_ nanofiber mats have been electrospun in a roll-to-roll system and collected on a substrate made of Polyethylene terephthalate (PET) nonwoven spunbond of 35 Grams per Square Meter (GSM). During the ES process, the flow rate of the solution loaded into the nozzle array was maintained at a constant value of 2.5 mL/h, whereas a voltage of 28 kV was applied to the needle’s nozzle arrays with a needle tip-collector distance equal to 13 cm.

### 2.2. Characterization Method

Scanning Electron Microscopy (SEM) and Energy Dispersive X-ray (EDX) analysis were performed on both PVDF-HFP and PVDF-HFP@TiO_2_ electrospun mats by means of a Zeiss Auriga (Zeiss, Oberkochen, Germany). To avoid beam damage, the surface sample was previously coated with ~20 nm of carbon by using a Quorum Q150T ES (Quorum Technologies, Ltd., Laughton, East Sassex, UK) sputter machine and further analyzed with an acceleration voltage operating at ~2 keV. To further confirm the reliability of the size distribution of the nanofiber electrospun membranes, Atomic Force Microscopy (AFM) measurements were also carried out on both the PVDF-HFP and PVDF-HFP@TiO_2_ samples. Unlike SEM analysis, the AFM sample preparation procedure does not require the application of an ultra-thin coating of electrically conducted material by means of the sputter machine. Therefore, a comparison between the AFM and the SEM analysis is fundamental to ensure that sputter coating does not drastically affect the morphology of both PVDF-HFP and PVDF-HFP@TiO_2_ electrospun membranes, thus avoiding possible overestimation of the real size of the nanofibers. The AFM measurements were carried out on the samples by means of a Cypher (Oxford Instruments, Oxford, UK) operating in tapping mode with a nominal cantilever elastic constant of 40 N/m. The obtained SEM and AFM images were analyzed and post-processed by using the open-source Gwyddion Software (http://gwyddion.net (accessed on 1 May 2023)). Raman spectroscopy measurements were also performed on the samples by means of high spatial resolution confocal Raman mapping (Renishaw Invia Confocal Raman microscopy, Glouchcestershire, UK) operating with a 532 nm green laser and recorded with a Peltier-cooled CCD detector. The spectra have been measured in the range between 100–1800 cm^−1^ with a spatial resolution of 1 cm^−1^.

### 2.3. Photocatalytic and Antibacterial Experiment

The photocatalytic disinfection of bacteria was investigated for PVDF-HFP@TiO_2_ electrospun under UV-A light exposure, by using the PVDF-HFP electrospun membrane as a control in the colony count method. Firstly, strains of *S. aureus* and *P. aeruginosa* were cultured in lysogeny broth (LB) medium and incubated at 37 °C for 24 h. After incubation, 2 μL of both *S. aureus* and *P. aeruginosa,* with a cell density of 10^8^ CFU/mL, were separately pipetted in 20 mL of deionized water (DI) and then mixed. These solutions have been further sprayed by means of nebulizers on the surface of the nanofiber mats made of PVDF-HFP and PVDF-HFP @TiO_2_, which were previously cut in different pieces of area equal to 1 × 1 cm^2^. The surfaces of the samples loaded with the two different bacterial solutions were further placed at 20 mm from a high-intensity UV-A light source (SST-10-UV, with a power of 875 mW/cm^2^, and peak wavelengths at 365 nm) and exposed to an irradiation time of 5 min. After the UV-A light exposure, these samples were immersed in 1 mL of LB and mixed for five minutes by means of a vortex mixer. After that, 50 μL of these bacterial dilutions, with an estimated cell density of around 10^4^ CFU/mL, were spread-plated on LB agar plates, and then incubated overnight (24 h) under a controlled growth temperature of 36 °C. Finally, the number of the bacteria grown on the plates for both PVDF-HFP and PVDF-HFP@TiO_2_ were counted and reported as *C*_0_ and *C*, respectively. These values were used to determine the photocatalytic efficiency of the PVDF-HFP@TiO_2_ membrane by means of the formula:

$$Antibacterial\;efficiency\;\left(\%\right)=\frac{C_{0}-C}{C_{0}}\;\times \;100$$

The photocatalytic activity of the PVDF-HFP @TiO_2_ electrospun membrane was also investigated by the photodegradation study of methylene blue (MB) under UV-A light source exposure. Nanofiber mats of both PVDF-HFP and PVDF-HFP @TiO_2_ (~30 cm^2^) were soaked in 5 mL of 2 ppm MB solution for 30 min in a dark environment to achieve adsorption–desorption equilibrium. The UV-A source (SST-10-UV, peak wavelengths at 365 nm) was kept at a distance of 20 mm from the top of the crystallizing dish containing the MB solution with the soaked mat, and the mixture was continuously stirred with a magnetic bar. The MB concentration was monitored over time by measuring the maximum absorption peak observed at ~664 nm with a PG Instruments T80+ UV/Vis spectrophotometer (using a glass cell of 1 cm path length, Leicestershire, UK). The photodegradation rate of MB (MB_t_/MB_0_) was therefore estimated considering the MB absorbance peak value (MB_t_) at a specific time (t) and the absorbance peak measured before UV-A irradiation (MB_0_) thanks to the linear relationship between absorbance and concentration within the range of the MB concentration chosen.

### 2.4. Filtration and Pressure Drop Testing Methods

Both Bacterial Filtration Efficiency (BFE) and Viral Filtration Efficiency (VFE) tests were performed on the nanofiber-based PVDF-HFP electrospun membrane and certificated by external companies such as Techno Analysis S.r.l. and Nelson Labs, according to the European standard (EN) 14683:2019 + AC:2019 and the American Society for Testing and Materials (ASTM) F2101, respectively. With regard to the BFE test, an aerosol of *S. aureus* with a culture suspension of about 1.7–3 × 10^3^ CFU and a mean particle size of (3.0 ± 0.3) μm was shot with a flow rate equal to (28.3 ± 0.2) L/min through a tested area of 50 cm^2^. The filtration test was replicated for five performances on five different tested samples, and for each of the tests the BFE was reported, in percentages, by the ratio of the difference between the average plate count total for the test control (*B*) and that measured for the sample (*T*) to the relative *B*. Therefore, the final BFE value of the PVDF-HFP electrospun membrane was estimated by the average value of the filtration tests obtained from the five independent replicas. The VFE test follows a similar procedure to that reported for the BFE test, but a suspension of a virus such as the bacteriophage ϕx174 is aerosolized. The aerosol, maintained between 1.1 and 3.3 × 10^3^ plaque-forming units (PFU) with a flow rate of around (28.3 ± 0.2) L/min and a mean particle size of (3.0 ± 0.3) μm, was delivered to a tested area of 40 cm^2^. As seen above for the BFE test, the final VFE value of the PVDF-HFP membrane was estimated as the average value of three independent filtration tests, and the filtration efficiency measured from a single replicate, which is expressed in percentages, was obtained by a comparison between the plate count recovered downstream with that observed from the positive control. Also, Particle Filtration Efficiency (PFE) and pressure drop tests were performed on the functionalized PVDF-HFP@TiO_2_ membrane from the Textal Materials and Machinery Research Group (TEMAG) at the Istanbul Technical University, according to the normative EN 149: 2001 [10]. The PFE value was measured by analyzing a tested area of 50 cm^2^, which was exposed to an aerosol of uncharged sodium chloride (NaCl) with a particle mean size of around ~0.6 μm at a constant flow rate of 95 L/min. This value is reported in percentages, as seen above for the BFE and VFE tests. Unlike the filtration test, the measure of the pressure drop allows us to obtain an estimate of the air flow resistance of the electrospun-based filter, and then the result will be a useful and common parameter to assess the breathability and the filtration performance for a mask. Differential pressure measurements (Δ*P*) were obtained for the PVDF-HFP@TiO_2_ electrospun membrane by comparing the downstream and upstream measurements for a tested area of 50 cm^2^, under a constant flow condition equal to 95 L/min.

### 2.5. Mechanical Properties

The tensile properties of the nanofiber-based PVDF-HFP@TiO_2_ electrospun membrane were evaluated with a Zwick/Roell Z010 (Ulm, Germany) equipped with a 1 kN load cell. The tests were performed on rectangular specimens with a length of 70 mm and a width of 10 mm using a test speed of 2 mm/min and a fixed grip-to-grip separation of 20 mm. The experiments were carried out at least in quadruple and the results were reported as mean values ± standard deviation.

### 2.6. Wettability Properties

The surface wettability of both the PVDF-HFF and PVDF-HFP@TiO_2_ electrospun membranes was determined by measuring the water Contact Angle (CA) by using an OCA 20 contact angle system (Data Physics Instrument GmbH, Filderstadt, Germany) at ambient temperature. The average CA value was obtained by measuring the CA on six different tested samples.

## 3. Results and Discussion

### 3.1. Characterization of the Nanofibrous Membranes

**Figure 1:**
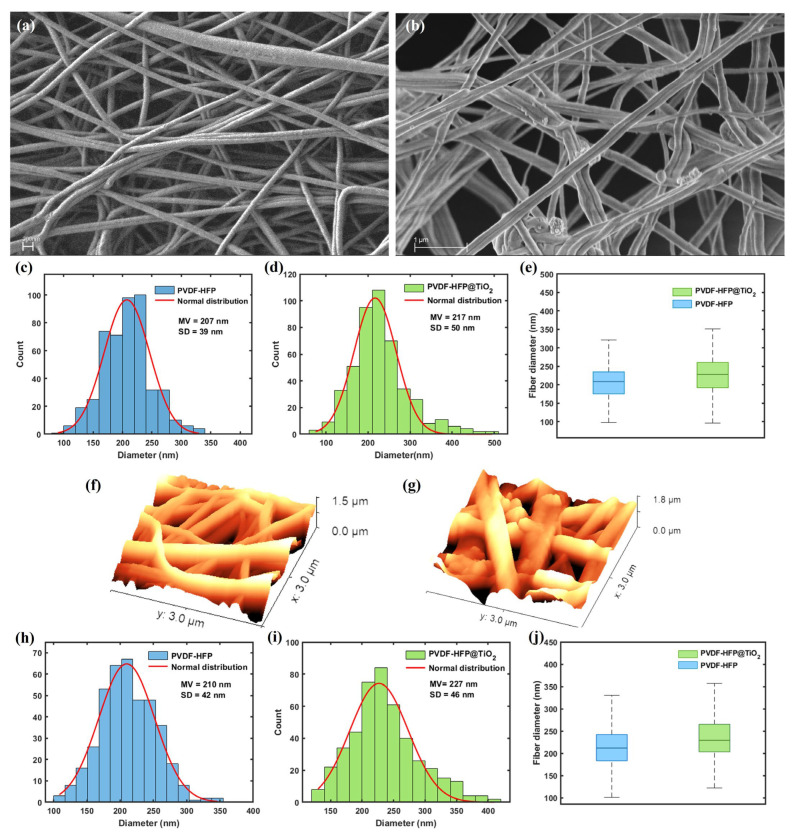
A comparison between the SEM micrographs for (**a**) PVDF-HFP and (**b**) PVDF-HFP@TiO_2_ electrospun membranes, both obtained at 40.00 kx. Nanofiber size distribution obtained by the statistical analysis performed on the SEM images acquired for (**c**) PVDF-HFP and (**d**) PVDF-HFP@TiO_2_, respectively; and relative (**e**) box plots, for a comparison. AFM images acquired for (**f**) PVDF-HFP and (**g**) PVDF-HFP@TiO_2_, respectively. Nanofiber size distribution obtained by the statistical analysis performed on the AFM images acquired for (**h**) PVDF-HFP and (**i**) PVDF-HFP@TiO_2_, respectively; and relative (**j)** box plots, for a comparison. For both the SEM and AFM techniques, the obtained distributions for PVDF-HFP and PVDF-HFP@TiO_2_ are statistically different (*p*-value < 0.05).

The SEM imagery performed on the PVDF-HFP electrospun membranes is reported in Figure 1a. The nanofiber mesh was observed to completely cover the support layer made of PET. Despite the formation of some fiber bundles, the structure of the single nanofibers proved to be continuous and smooth, thus involving a large surface-to-volume ratio and then a higher nanofiber packing density per unit area compared to the larger PET-based microfibers (Figure S1a). Therefore, the concentration obtained with high molecular weight PVDF-HFP prepared at 16 wt.% was shown to promote a suitable polymer entanglement in the solution process, leading to the formation of uniform nanofibers with a low number of beads defects (Figure S1b) [55]. Indeed, the addition of acetone in the spinning solution of PVDF-HFP has been reported in the literature to significantly suppress the formation of beads for concentrations ranging between 15% and 20%, thus influencing the quality of the resulting nanofibers [56]. Also, it led to a decrease in the high surface tension in the mixed spinning solution, thus preventing the polymer jet from breaking into individual droplets and allowing for the formation of uniform nanofibers during the ES process [55,57,58].

**Figure 2:**
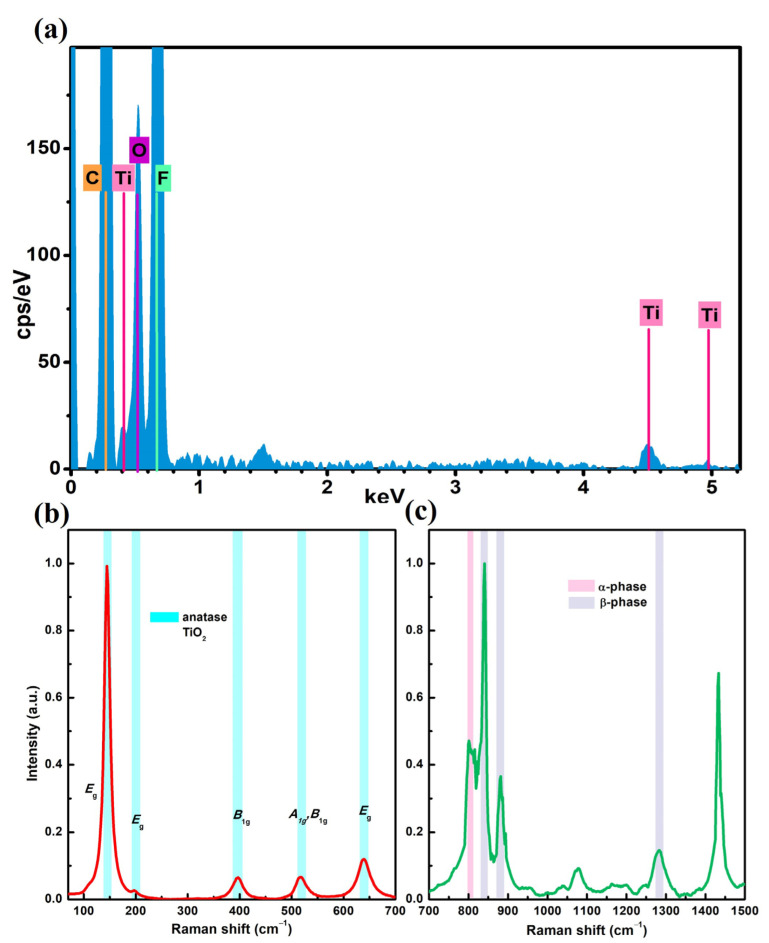
(**a**) EDX spectrum and Raman spectra (**b**,**c**) performed on the PVDF-HFP @TiO_2_ electrospun membrane; these last show the active mode contributions due to the presence of (**b**) anatase TiO_2_ NPs and (**c**) PVDF-HFP-based nanofiber polymer. Both the Raman spectra have been normalized to one at maximum peak.

Morphological changes occurring at the nanofiber surface level were observed on PVDF-HFP @TiO_2_ due to the inclusion of TiO_2_ NPs in the precursor solution. The high-resolution SEM images performed on the functionalized electrospun membrane show that agglomerates of TiO_2_ NPs were tightly adsorbed and distributed along the nanofibers’ surfaces (Figure 1b). Because of the low concentration of TiO_2_ NPs, these agglomerates appear sparse on the single nanofibers with different sizes. However, they are distributed homogeneously on the electrospun membranes (Figure S2a,b). In addition, from a comparison between the analysis of the SEM images carried out on both the untreated and functionalized electrospun-based membranes reported in Figure 1c,d, it can be seen that the diameter size of the PVDF-HFP polymer-based nanofibers tends to increase from (207 ± 30) nm to (217 ± 50) nm, as a consequence of the addition of TiO_2_ NPs in the precursor spinning solution. This shift to a higher size was also observed from the analysis performed on the AFM topographical measurements (Figure 1f,g), which provided a mean value equal to (210 ± 42) nm and (227 ± 46) nm for the PVDF-HFP and PVDF-HFP @TiO_2_ electrospun membranes, respectively (Figure 1h,i). The Welch two-sample *t*-tests performed on the relative nanofiber distributions showed that a significant difference occurred between the mean values for the PVDF-HFP @TiO_2_ and the PVDF-HFP obtained from both the SEM (Figure 1e) and the AFM (Figure 1j) analysis, thus indicating that modification of the nanofiber size is most likely induced by the functionalization process. This fact can be linked to the capacity of the TiO_2_ NP_S_ to involve a variation in both the viscosity and conductivity properties of the polymer jet. This latter, during the ES process, can undergo stronger bending instability under high voltage and lead to the formation of more coarse nanofibers [59,60,61,62,63]. In order to analyze the chemical characterization of the functionalized membranes, EDX analysis was conducted on a given micro area of the SEM image. The measured EDX spectrum showed both the elements of the composite nanofibers (Figure 2a).

The intense peaks for C and F elements observed at 0.27 and 0.68 keV, respectively, can be attributed to the CF_2_ groups, forming the chemical structure of the PVDF-HFP electrospun nanofiber mats. Meanwhile, the low peaks centered at 0.4, 4.5, and 4.9 keV can be ascribed to Ti, thus confirming the presence of TiO_2_ NPs on the nanofiber electrospun membrane. The low signals measured for the Ti element were expected, since a low concentration of TiO_2_ NPs was mixed in the polymer solution to functionalize the PVDF-HFP-based electrospun membrane [64,65,66]. On the other hand, the further investigation of the Raman spectra showed intense bands at a low frequency range around 145 (*E_g_*), 199 (*E_g_*), 396 (*B_1g_*), 518 (*A_1g_* and *B_1g_*), and 639 cm^−1^ (*E_g_*) due to the Raman active mode of the anatase [67,68] (Figure 2b). Meanwhile, the other peaks observed at 840, 881, 1279 cm^−1^, and 796 cm^−1^ can be ascribed to the β and α crystal phase of the PVDF nanofibers (Figure 2c and Table S1) [69,70]. The analysis carried out shows that despite the addition of TiO_2_ NPs in the precursor spinning solution leading to a slight increase in the nanofiber diameter, these nanostructures are evenly dispersed on the PVDF-HFP @TiO_2_ membrane during the ES nanofiber process. The good dispersion of agglomerates on the fiber surface is advisable on the electrospun membranes, since it can improve the contact with both pathogens and aerosol contaminants, thus leading to a higher interception and further photocatalytic deactivation of potential bacteria and viruses [8].

### 3.2. Antibacterial Test

**Figure 3:**
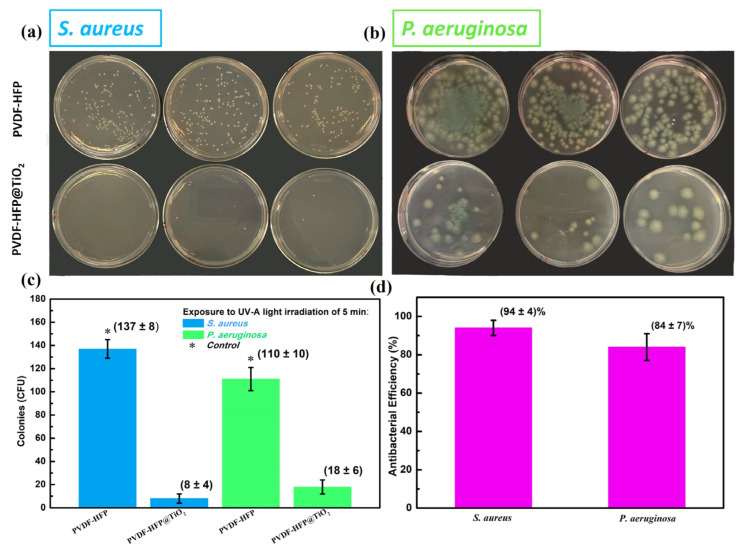
Photo images of plate incubation for (**a**) *S. aureus* and (**b**) *P. aeruginosa* after 5 min of UV-A treatment for the PVDF-HFP and PVDF-HFP @TiO_2_ electrospun membranes; a drastic reduction is shown in both bacteria, when incubated with the photocatalytic PVDF-HFP @TiO_2_ electrospun membrane. (**c**) CFU count of the respective plates and further estimation of the (**d**) antibacterial efficiency for the PVDF-HFP @TiO_2_ electrospun membrane.

The photoinactivation of *S. aureus* and *P. aeruginosa* bacteria was investigated by exposing both the untreated PVDF-HFP and the functionalized PVDF-HFP @TiO_2_ membranes to a UV-A light source. In order to ensure the reproducibility of the antibacterial test, the photocatalysis studies were performed three times and, for each one, three replicates on plates were made. For comparison, Figure 3, panel (a) and (b), displays examples of replicates of Petri dishes obtained by a single experiment carried out for both the untreated and functionalized electrospun membranes in the presence of the tested bacteria, after 5 min of UV-A light exposure.

The Petri dishes collected for the control PVDF-HFP membrane display a large number of colonies for both the tested *S. aureus* and *P. aeruginosa* bacteria compared to those observed on the plates collected for the relative PVDF-HFP @TiO_2_ membranes. Indeed, the comparison between the values of the average number of colonies for the three independent experiments, which are obtained by an estimate of the surviving colonies counted for both the bacteria tested in the respective agar Petri dishes collected for the untreated and the functionalized electrospun membranes, clearly indicates that the incorporation of photocatalytic TiO_2_ NPs on the electrospun-based membrane induced a high inactivation rate after a short exposure time to a UV-A light source (Figure 3c). Furthermore, the antibacterial performance estimated from the count of the average number of colonies indicates that the photocatalysis process is more efficient in deactivating *S. aureus* than *P. aeruginosa* bacteria (Figure 3d). Both *P. aeruginosa* and *S. aureus* cause infections recalcitrant to many antibiotic treatments and are multi-drug resistant [71]. Moreover, *P. aeruginosa* is also known for possessing intrinsic resistance mechanisms against adverse conditions and also for developing new ones quickly, by mutations or by horizontal gene transfer [54]. Therefore, it is probable that its strong metabolic versatility confers on it a higher ability to survive in UV-A exposure [72]. Experimental evidence showing a significant antibacterial activity against both *S. aureus* and *P. aeruginosa* strains due to the activation of the photocatalytic properties of TiO_2_ NPs under different time exposures to a UV-A source have been reported in the literature [73,74]. Some studies showed that the *S. aureus* strain is more resistant to the photocatalytic oxidation of the inner plasma membrane induced by ROS formation than the *P. aeruginosa* strain [73,75]. Such different photoinactivation behavior has been ascribed to the characteristic structure of the relative bacterial cell walls. The outer membranes of gram-positive bacteria, such as *S. aureus*, are composed of a thick layer of peptidoglycans (PG) (20–80 nm), which need a longer time for ROS to penetrate and damage the inner plasma membrane causing the cell’s death [75]. Unlike gram-positive bacteria, gram-negative strains such as *P. aeruginosa* have a thin overall membrane (~10 nm), which is characterized by an external layer composed of lipopolysaccharide (LPS) and an inner one of PG, which results in a less effective protection from oxidative stress. However, other investigations showed that the photocatalytic inactivation for gram-negative bacteria was comparable to that measured for gram-positive, but only for longer exposure times of TiO_2_ active compounds to the UV-A source [76]. Also, in the work carried out by R. J. Barnes et al., it was observed that *S. aureus* was more sensitive to the photocatalytic activity of TiO_2_ NPs as a function of its concentration compared to *P. aeruginosa,* within the same time of exposure to the UV-A source [74]. Moreover, a clear difference in photocatalytic inactivation was also observed between the gram-negative *E. coli* and *P. aeruginosa* bacteria, thus suggesting that characteristic properties of the outer relative cell’s membrane do not strongly influence the ability of ROS to affect the cell viability. These studies therefore indicate that the cell viability of both *P. aeruginosa* and *S. aureus* can be drastically affected by the UV-A-induced photocatalytic activity of the TiO_2_ compound by the generation of ROS, but their response to this bactericidal process can occur with different kinetic mechanisms and can be dependent on other experimental parameter conditions given, for instance, by the light intensity source, nanoparticle aggregation, and crystal state of TiO_2_ [61,73,74]. However, recent studies showed that a longer exposure of photocatalytic TiO_2_ NPs to UV–visible light sources leads to a complete bacteria disinfection efficiency in the functionalized polymer fiber membrane, regardless of the bacteria species. Li et al, experimentally observed that the designed electrospun filter based on PVA, PEO, and cellulose nanofibers containing a low dose of TiO_2_/N-TiO_2_ (~29.7 mg/cm^3^) NPs provides excellent antibacterial activity for both *E. Coli* and *S. aureus* (~100%), after a visible–UV light exposure time of ten minutes [22]. In the work carried out by Chen et al., a high inactivation rate of at least ~99% against *S. aureus* was obtained by exposing the PAN-based electrospun filter functionalized at 2 wt.% TiO_2_ NPs to 30 min of UV irradiation [38]. Also, since a significant resistance of *Coliforms* bacteria to UV-A-activated TiO_2_ NPs was observed compared to *E. coli* in the contaminated PVDF-TiO_2_ fiber membrane, a longer exposure to a UV-A source (~10 min) was required to achieve a complete inactivation of both the bacteria [77]. These studies indicate that a higher exposure time to UV-A light can be beneficial to achieve a better antibacterial performance against both gram-negative and gram-positive bacteria strains, especially for the more resistant ones, in the functionalized electrospun membranes, thus reducing the risk of disease spread, which is caused by the handling and reuse of contaminated face masks.

**Figure 4:**
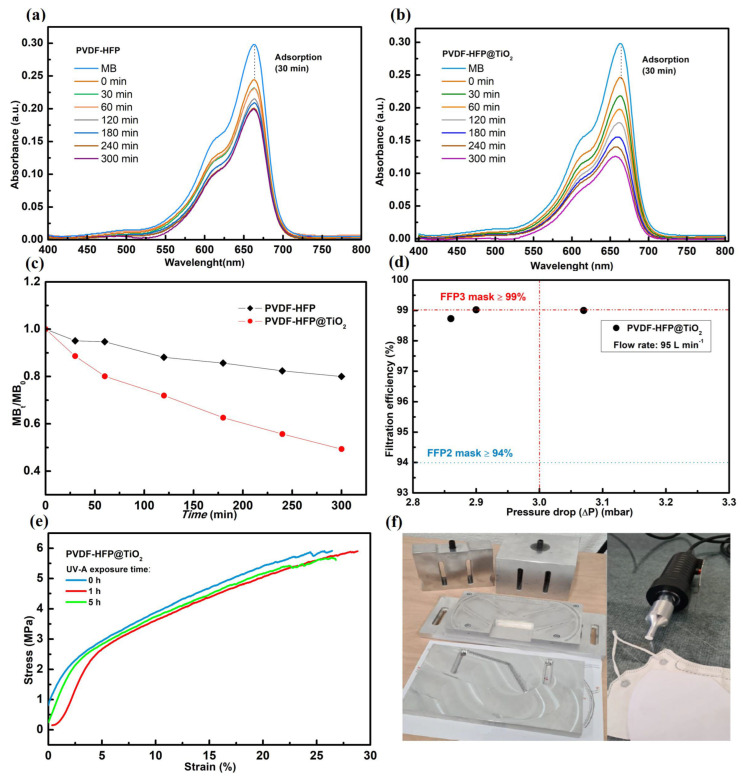
UV-A light dependence of the UV–visible absorbance spectra collected for the PVDF-HFP (**a**) and PVDF-HFP @TiO_2_ (**b**) electrospun membranes. Time-dependent MB degradation curves (**c**). Filtration efficiency values measured from the PFE test carried out on the functionalized electrospun membrane (**d**). A comparison between the tensile-stress curves (**e**) measured for the PVDF-HFP @TiO_2_ electrospun membranes after 0, 1, and 5 h of UV-A exposure time. A picture showing the fabrication of a face mask (**f**) made by including the PVDF-HFP @TiO_2_ electrospun membrane as a filter.

The effectiveness of the photocatalytic PVDF-HFP @TiO_2_ membrane in promoting the formation of ROS under exposure to a UV-A source has been assessed by the photodegradation study of MB. The UV–vis absorption spectra measured for the MB solution prepared at 2 ppm before and after UV-A light irradiation for the respective untreated and functionalized electrospun membranes are shown in Figure 4a,b. In the absence of UV-A light, a decrease in the absorbance peak at 664 nm was observed for both the PVDF-HFP and PVDF-HFP @TiO_2_ electrospun membranes, thus suggesting an adsorption capacity of the polymer-based electrospun membranes due to a high specific surface area [78,79]. With the occurrence of UV-A irradiation, the reduction in MB concentration was higher in presence of the PVDF-HFP @TiO_2_ membrane than for PVDF-HFP. Moreover, the further shift of the peak down to low frequency (blueshift) clearly indicates that demethylation of MB occurs concurrently with the formation of radical species (such as hydroxyl •OH), because of the photo-induced formation of electron–hole pairs in the TiO_2_ NPs-embedded electrospun membrane [78,80,81]. Figure 4c shows that the MB degradation rate obtained for the PVDF-HFP electrospun membrane remained quite stable at around 20% after 5 h of UV-A light exposure, whereas a monotonous decrease occurred in the presence of the PVDF-HFP @TiO_2_ membrane with a dye degradation after 5 h of treatment equal to 51%. This different behavior of MB removal over time reflects the fact that the TiO_2_ NPs present on the nanofiber surface promote the formation of electron–hole pairs under UV-A irradiation, thus resulting in the production of ROS [61,79,82,83,84,85]. The obtained results suggest that the generation of ROS induced by the interaction among the UV-A light and the TiO_2_ NPs-embedded PVDF-HFP-based electrospun membrane involves the high photoinactivation of both gram-positive and gram-negative bacteria at the fiber surface level. In contrast, most of the bacteria tested on the untreated PVDF-HFP remained alive in both cases, because of the limited antibacterial efficiency caused by the exposure of only the electrospun membrane to the UV-A source.

### 3.3. Filtration Performance

In order to evaluate the application of PVDF-HFP@TiO_2_ as a filter for the manufacturing of advanced face masks, measurements of the bacterial, viral, and particle filtration efficiency as well as a breathability test were performed on the untreated and functionalized electrospun membranes, according the EN 14683:2019, ASTM F2101, and EN 149 standards, respectively. With regard to the BFE, all the values obtained from each of the five independent tests were significantly higher (~99%) than the limit required from the EN 14683:2019 standard, which is set to 95%, thus indicating that the PVDF-HFP electrospun membrane provides a suitable grade of protection from the penetration of aerosol carriers for *S. aureus* bacteria. The VFE tests were performed to assess, on the other hand, the resistance of the electrospun-based PVDF-HFP to the penetration of the ϕx174 bacteriophage, an envelope virus with a similar structure to that present in SARS-CoV-2 [86]. Two out of the three tests performed showed VFE values higher than 99.9%, while the third result was about 99.8%, therefore providing for the PVDF-HFP electrospun membrane an overall performance comparable with those reported for any commercial mask such as the N95 FFRs [87,88]. Previous investigations on either the bacterial or virus filtration performance for PVDF-based electrospun membranes have been reported in the literature [89,90,91,92]. In a recent study, Shen et al. demonstrated that PVDF electrospun membranes characterized by a fiber diameter of around ~300 nm provided a higher capture of an aerosol solution based on Murine hepatitis virus A59 (MHV-A59), a β-coronavirus strain with a similar structure size to SARS-CoV-2 (size ~85 nm), compared to that observed for commercial filters with a larger fiber size [91]. Also, in the work carried out by Felix Swamidoss et al., it was observed that a decrease in both fiber diameter and interstitial space between the fibers due to the addition of TiO_2_ nanotubes at different concentrations in the precursor solution led to an improvement in both BFE and air permeability for the PVDF electrospun membrane [89]. Since the size of the submicron particles carried by the aerosol can be comparable with the nanofiber diameter, a slip flow regime occurs at the single nanofiber level, involving a high capture of particles due to the interception mechanism [93,94,95]. In addition, a reduction in the drag force friction involved in this regime creates a low-momentum exchange between the submicron particles and the nanofiber surface, resulting in a low pressure drop for the electrospun membrane. Therefore, an optimization of both fibers and inter-fiber space in PVDF-based electrospun membranes may result in the effective interception of aerosol carriers of either bacteria or viruses, while providing a low pressure drop with a proper breathability [26,89,90]. Figure 4d reports a comparison between the filtration performance of the PVDF-HFP electrospun membrane functionalized with TiO_2_ NPs at 2 wt.% and those required for the FFPs, according to the standard EN 149:2001 + A1 2009. The obtained results show that the PVDF-HFP @TiO_2_ provides an excellent PFE value, which is higher than that required for FFP2 (≥94%) and in accordance with the minimum conditions required for FFP3 face masks to ensure a high-level efficiency in submicron particle removal (≥99%). Also, the relative pressure drops measured were found in the three-test to be comparable to or lower than the limit value required for the FFP3 (~3.0 mbar), thus indicating that a low air flow resistance occurs in the fabric, making breathing comfortable for the wearers. Several studies reported that a positive correlation occurs between BFE and PFE, and a slight difference observed in the assessment of the filtration performance of face masks between these two methods is mainly due to the utilization of a different aerosol size distribution [87,96,97]. The aerosol generated for the PFE test is composed of monodisperse particles, which are characterized by a diameter of ~0.6 μm. Unlike PFE protocol, the mean particle size of ~3.0 μm imposed by the BFE method is obtained as an average estimate of the total count of the six stages of the aerosol cascade impactor, which is characterized by a wide granulometric range of 0.6–7 μm. Since all particles within this range are considered in the estimation of the BFE, a transmission of particles with sizes below 3.0 μm, even if they are a small amount of the total generated aerosol, through the tested filter can create a significant decrease in the BFE [96,97]. It is important to note that the value of BFE measured for the PVDF-HFP membrane was equal at least to ~99%, thus indicating a constant filtration through all the granulometric range, and also for particles smaller than 3.0 μm. This value is therefore comparable with that of the PFE measured for the PVDF-HFP@TiO_2_ electrospun membrane, thus suggesting that the functionalization of the PVDF-HFP with the addition of a low dose of TiO_2_ NPs does not affect the high filtration performance in removing aerosol contaminants. Although a possible concern with using a high amount of TiO_2_ NPs in filtering devices could arise from their toxicity, the release mechanisms of these NPs and their aggregates from face masks are currently unknown and there are no available standardized methods to determine whether or not they are released during common use [98,99]. In a recent study, Eveline Verleysen et al. experimentally observed that since most of the TiO_2_ NPs remain inside the fiber matrix during the filament production, only those located at the fibers’ surface, which was estimated to be only a fraction, ranging between 2–9%, are assumed to be released during the prolonged wearing of face masks [98]. However, the maximum amount of TiO_2_ NPs loaded in an effective area of about 166 cm^2^ of the PVDF-HFP@TiO_2_ electrospun nanofiber filter was estimated from the initial precursor solution to be around ~260 μg, which is up to three times lower compared to the minimum amount detected for some single-use and reusable face masks available on the market [98]. Indeed, the functionalized electrospun membrane is characterized by a lower basis weight (~0.8 GSM) compared to that of commercial filters, thus resulting in the presence of a thin layer above the PET spunbond layer (Figure S3) with a consequent reduction in the presence of TiO_2_ NPs [100]. Additionally, cytotoxicity studies of TiO_2_ NPs proved that the cell viability of several human cell lines of diverse origin, such as lung cells (A549), liver cells (HepG2), and neurons (SH-SY5Y), remained quite stable at above 60%, including after prolonged exposure to higher concentrations than those expected to be released at the worst from the fiber surface of the PVDF-HFP@TiO_2_ during continuous breathing (~23 μg) [101,102,103,104]. These analyses were in accordance with what was observed in cytotoxicity assays performed on several functionalized electrospun-based membranes, where the cell proliferation of the fibroblasts L929 and A549 proved not to be drastically affected by the presence of TiO_2_ on the electrospun mats [105,106,107]. Although further research will be needed to regulate the quality parameters to validate the risk of NPs on face masks, these results suggest that the low dose of TiO_2_ NPs embedded in the electrospun filter matrix is negligible and could not provide a high risk of tissue damage. Moreover, focusing on the tensile properties, both the tensile strength and modulus of the PVDF-HFP @TiO_2_ membrane (Table S2) proved to be almost unaffected by prolonged UV-A light exposure for 1 h and 5 h. In particular, their values remain quite stable at around 6.2 MPa and 100.0 MPa, respectively, thus demonstrating their low tendency to degrade over a prolonged irradiation process (Figure 4e). In the literature, photocatalytic degradation tests of contaminated waters revealed a high stability of the PVDF-TiO_2_ electrospun membrane after a long exposure time to UV radiation [34,36,108,109,110]. The photocatalytic performances of these functionalized PVDF-based electrospun membranes were observed to remain quite stable after several repeated cycles under UV radiation in a water contaminant, thus indicating that TiO_2_ NPs were tightly embedded on the fiber surface, as a consequence of the ES process [34,109,110]. Because of the fast response obtained in the remotion of the bacterial contaminant under a short exposure time to a UV-A source, the potential release of TiO_2_ NPs from the PVDF-HFP@TiO_2_ electrospun filter can be significantly low. Therefore, its implementation in face masks can be advantageous to reduce the risk of NPs released into the environment, which are observed in disposable commercial masks due to the long exposure time to standard disinfection methods [111]. In addition, from a comparison between the measurements of the Water Contact Angle (WCA) performed on both the PVDF-HFP and PVDF-HFP@TiO_2_ and reported in Figure S4, it can be seen that the addition of a low dose of TiO_2_ NPs leads to a slight increase in the surface hydrophobicity in the functionalized electrospun membrane. This improvement in surface wettability can be effective in preventing the deposition of droplets which can be propelled by intense coughs and sneezes, thus decreasing not only the penetration of biological contaminants, but also their proliferation on the photocatalytic filter device [22,112,113,114,115]. The results found here therefore indicate that the PVDF-HFP-based electrospun membrane optimized with a low concentration of TiO_2_ NPs can be beneficial as filter media for the manufacturing of advanced face mask devices due to both the high submicron particle efficiency with optimal pressure drop and for the bactericidal activity through photocatalytic activation under UV-A exposure. The fabrication cost of the photocatalytic electrospun filters is also economic and easily available on a commercial scale [116]. As shown in Figure 4f, a three-layer face mask device has been designed by means of the ultrasonic sewing technique, by including the PVDF-HFP @TiO_2_ membrane as filter media and a third spunbond layer made of PP, used as inner mechanical support. The PVDF-HFP@TiO_2_ sandwiched between the two spunbond fabrics acts as a filtering system, without coming in direct contact with the human face. This avoids any possible risk due to skin irritation as well as minimizing the possibility of having a direct release of NPs from the cloth during wearing. An increase in the manufacturing of photocatalytic electrospun nanofibers can be achieved by employing a multi-nozzle ES setup for an industrial line equipped with an automated unwinding and rewinding system [117]. The roll-to-roll automation system can adjust the winding speed of the roll during the ES process, thus ensuring a continuous and uniform production of the functionalized nanofibers on the PET substrate support sheets, with a width around 1 m (Figure S5). Hence, the utilization of automated and programmable multi-nozzle ES setup machines with high throughput rates would make the manufacturing of functionalized nanofiber-based filters advantageous in applications for self-sterilizing wearable devices on a large scale. This would lead to a wider utilization of this high-performance face mask, thus limiting the risk of bacteria proliferation due to handling and to the prolonged use of commercial face masks.

## 4. Conclusions

The aim of this research was to provide evidence that ES is an easily upscaled method to design advanced electrospun filters with promising properties in terms of both submicron particle filtration and antibacterial performance. Such properties can help in dealing with the present issues originating from the outbreak of the COVID-19 pandemic, given the contamination from bacteria on PPE surfaces due to the reusability of disposable face masks. This study showed that the electrospun PVDF-HFP obtained by means of multi-nozzle ES exhibits a suitable filtration efficiency toward S. *aureus* (BFE ~99%) and ϕx174 bacteriophage (VFE > 99.8%) in accordance with the EN 14683:2019 and ASTM F2101, respectively. This suggests its feasibility as a filter device in preventing bacterial and viral infections from possible airborne contaminants. The observed low-diameter size of the nanofiber (~207 nm) leads to a significant improvement in the mechanical adsorption of fine particles, thus increasing the possibility of pollutant deposition on the nanofiber surface. We observed that the incorporation of TiO_2_ NPs at 2 wt.% in the polymer precursor solution created a significant antibacterial efficiency in the PVDF-HFP electrospun membrane against *S. aureus* (~94%) and *P. aeruginosa* (~85%), under a short exposure time to UV-A irradiation. Indeed, the analysis carried out for PVDF-HFP@TiO_2_ revealed that the photoactivation of the TiO_2_ NPs embedded at the nanofiber surface promotes a suitable deactivation of these bacteria, by generating ROS. In addition, the functionalized filter media provide satisfying mechanical properties, i.e., a tensile modulus of 101 MPa and a tensile strength of 6.2 MPa, and a high filtration efficiency in the removal of PM_0_._6_ particles (~99%) with an optimal pressure drop of around 3 mbar, according to the EN 149: 2001 standard. Such customizability obtained by means of the multi-nozzle ES enables an interesting perspective on the rapid manufacturing of low-cost high-filtering electrospun-based devices on an industrial scale.

## Data Availability

Data are contained within the article and Supplementary Materials.

## Supplementary Materials

The following supporting information can be downloaded at: https://www.mdpi.com/article/10.3390/polym15234586/s1, Figure S1: SEM micrographs showing the electrospun PVDF-HFP-based nanofiber mats on spunbond-based PET; Figure S2: SEM micrographs of PVDF-HFP and PVDF-HFP @TiO_2_, both obtained at low magnification; Table S1: band assignment for the Raman spectra of the PVDF-HFP@TiO2 electrospun membrane. Figure S3: SEM micrographs showing a comparison between the thickness of the electrospun PVDF-HFP @TiO_2_ with that of the commercial spunbond PET; Table S2: a comparison between the mechanical characteristics, such as tensile strength and elongation at the break, obtained from the analysis of the stress–strain curves carried out on the PVDF-HFP @TiO_2_ electrospun membranes, after different UV-A exposure times; Figure S4: photographs showing a single water drop on PVDF-HFP and PVDF-HFP @TiO_2_ electrospun mats, with the related average CA values; Figure S5: photo images of a PVDF-HFP @TiO_2_ electrospun membranes mats produced by means of a high-throughput multi-nozzle Electrospinning machine for industrial production.
