# Supplementary material for: Upscaling of Electrospinning Technology and the Application of Functionalized PVDF-HFP@TiO2 Electrospun Nanofibers for the Rapid Photocatalytic Deactivation of Bacteria on Advanced Face Masks
Source: Polymers (Basel). 2023 Nov 30;15(23):4586. doi: 10.3390/polym15234586 (PMC10708761; doi:10.3390/polym15234586)
Supplement: Supplementary file 1 [file polymers-15-04586-s001.zip › polymers-2674184-supplementary.pdf]

Upscaling of Electrospinning technology and application of functionalized PVDF-HFP@TiO_2 electrospun nanofibers for rapid photocatalytic deactivation of bacteria on advanced face masks

Adriano Cimini[1,2*], Alessia Borgioni[3], Elena Passarini[3], Chiara Mancini[1], Anacleto Proietti[1], Luca Buccini[1], Eleonora Stornelli[1], Emily Schifano[3], Simone Dinarelli[4], Francesco Mura[1,5*], Claudia Sergi[6], Irene Bavasso[6], Barbara Cortese[7], Daniele Passeri[1,5], Enrico Imperi[2], Teresa Rinaldi[3], Alfredo Picano [8] and Marco Rossi[1,5]

[1]Department of Basic and Applied Sciences for Engineering, Sapienza University of Rome, 00161 Rome, Italy
[2]LABOR s.r.l., Industrial Research Laboratory, Via Giacomo Peroni, 386, Rome, Italy
[3]Department of Biology and Biotechnology, "Charles Darwin", Sapienza University of Rome, 00185 Rome, Italy
[4]Institute for the Structure of Matter (ISM), National Research Council (CNR), 00133 Rome, Italy
[5]Research Center for Nanotechnology for Engineering of Sapienza (CNIS), Sapienza University of Rome, 00185 Rome, Italy
[6] Department of Chemical Engineering Materials Environment, Sapienza University of Rome & UdR INSTM, 00184 Rome, Italy
[7] National Research Council-Institute of Nanotechnology (CNR Nanotec), c/o Edificio Fermi, Sapienza University of Rome, 00185 Rome, Italy
[8] National Research Council of Italy, Institute for Microelectronics and Microsystems (CNR-IMM), Via Piero Gobetti 101, 40129 Bologna, Italy
*Correspondence: adriano.cimini@uniroma1.it; francesco.mura@uniroma1.it

Supplementary Information

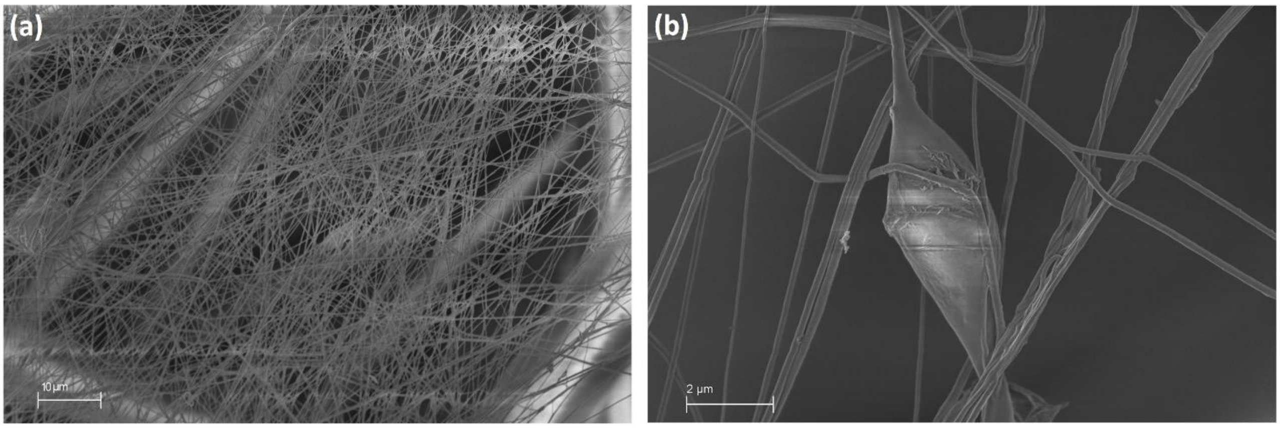

Figure S1. SEM micrograph showing the electrospun of (a) PVDF-HFP-based nanofiber mats on spunbond-based PET obtained at 3.00 k x. SEM image displaying the formation of single bead (b) at 21.00 k x.

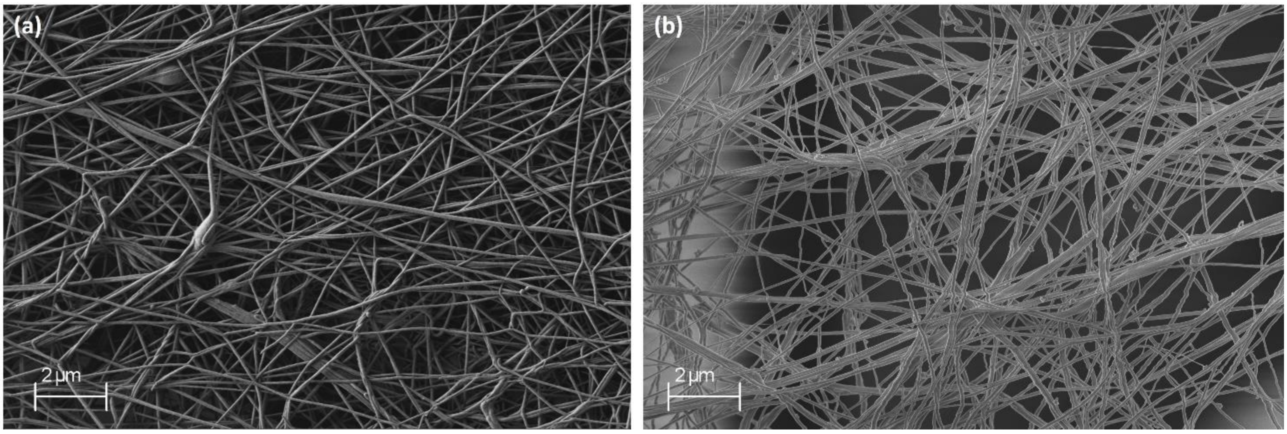

Figure S2. SEM micrograph showing the electrospun of (a) PVDF-HFP and (b) PVDF-HFP@TiO_2-based nanofiber mats, both obtained at 10.00 k x.

Table S1

Band assignment for the Raman spectra of PVDF-HFP@TiO2 electrospun membrane.

Raman shift (cm^{-1})	Assignment
145	E_g
199	E_g
396	B_{1g}
518	A_{1g} and B_{1g}
639	E_g
796	CH_2 rocking
840	CH_2 and CF_2 rocking and asymmetric stretching
881	CC and CF_2 rocking and asymmetric stretching
1279	CF_2 and CC asymmetric and symmetric stretching

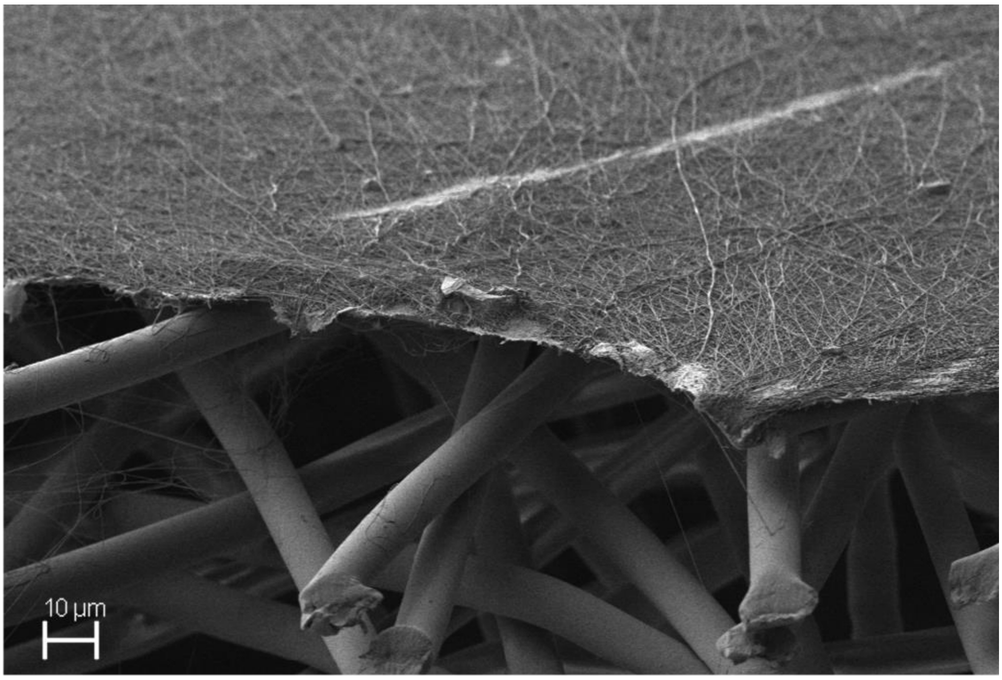

Figure S3. SEM micrograph obtained at 1.00 k x showing the difference in thickness between the thin electrospun layer of PVDF-HFP@TiO_2-based nanofiber mats and the thick commercial one with spunbond-based PET.

Table S2
A comparison between the mechanical characteristic, such as tensile strength and elongation at the break, obtained from the analysis of the stress–strain curves carried out on the PVDF-HFP @TiO_2 electrospun membranes, after different UV-A exposure time.

UV-A exposure time (h)	Tensile Strength (MPa)	Strain at maximum stress (%)
0	6.2 ± 0.3	28 ± 2
1	6.6 ± 0.6	35 ± 2
5	6.1 ±0.4	31 ± 4

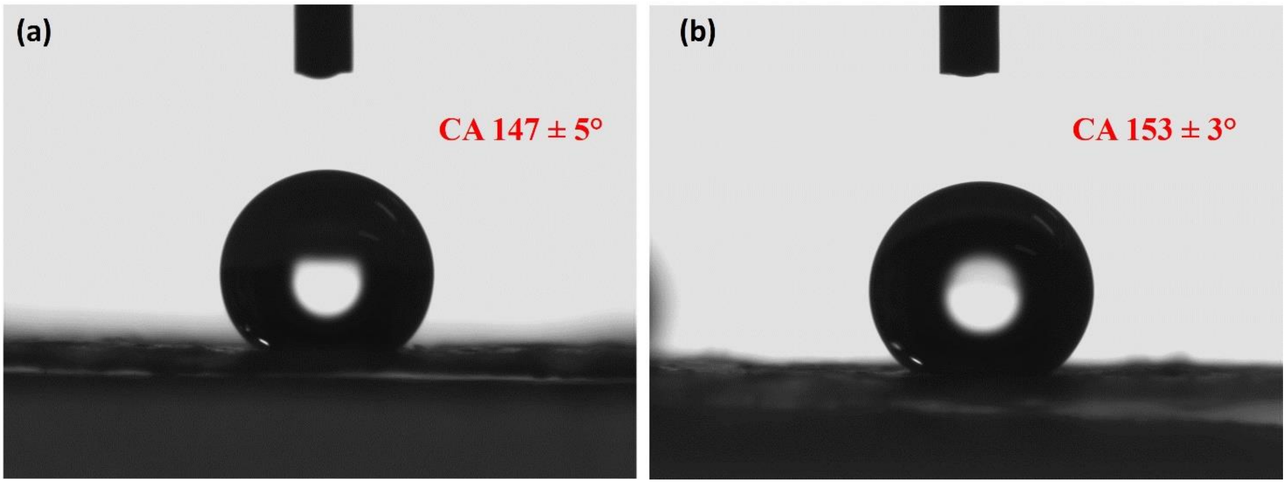

Figure S4. A comparison between the contact angles (CA) measurements performed for both the (a) PVDF-HFP and (b) PVDF-HFP@TiO_2 electrospun mats.

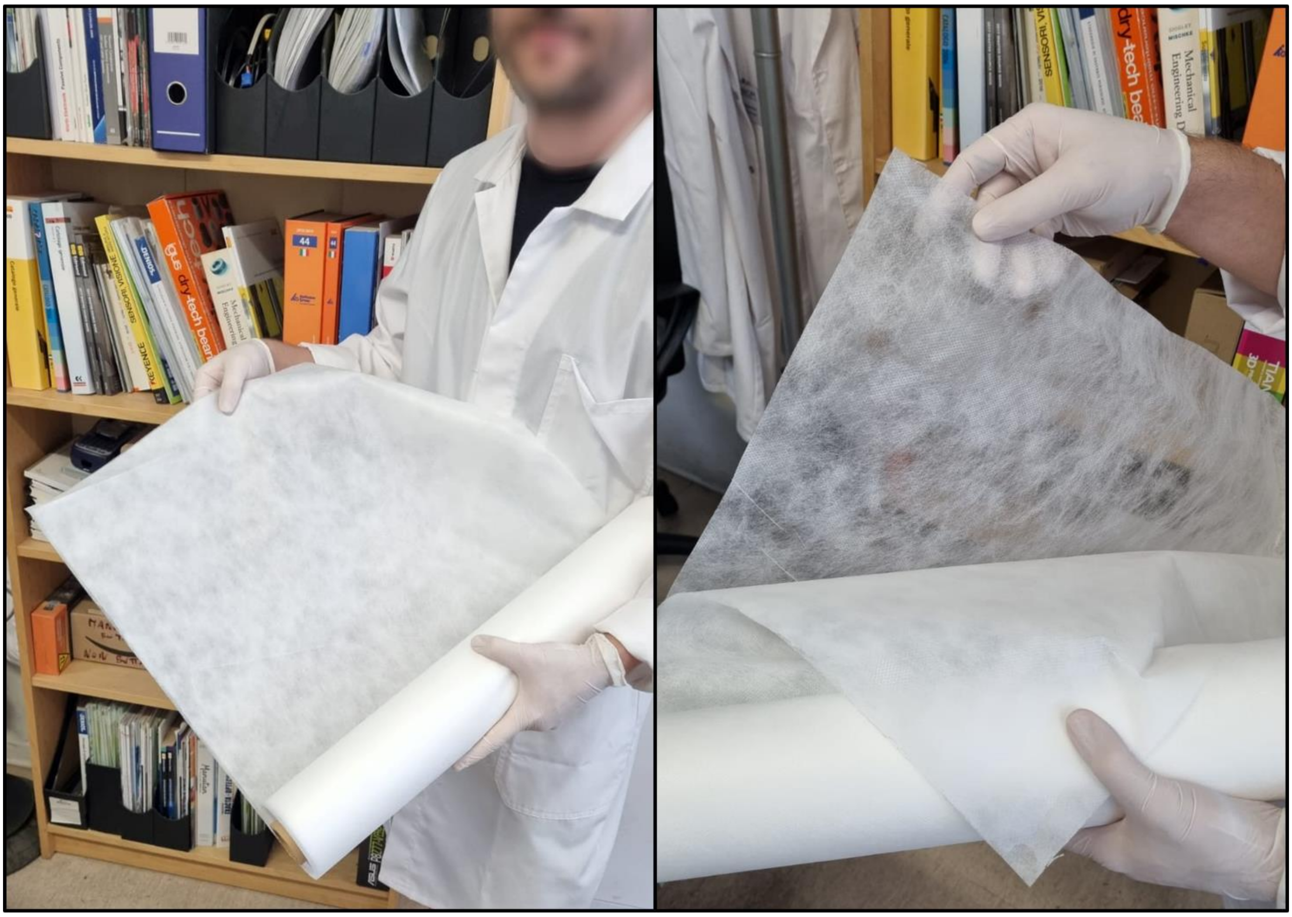

Figure S5. Photo images of a PVDF-HFP @TiO_2 electrospun membranes mats (left) produced by means of a high-throughput multi-nozzle Electrospinning machine for industrial production. The image on the right shows the nanofibers electrospun on PET spunbond layer.
